# CT-based radiomics signature for differentiation between cardiac tumors and thrombi: a retrospective, multicenter study

**DOI:** 10.1038/s41598-022-12229-x

**Published:** 2022-05-17

**Authors:** Ji Won Lee, Chul Hwan Park, Dong Jin Im, Kye Ho Lee, Tae Hoon Kim, Kyunghwa Han, Jin Hur

**Affiliations:** 1 Department of Radiology, Pusan National University Hospital, Pusan National University School of Medicine and Medical Research Institute, Busan, South Korea; 2 Department of Radiology, Gangnam Severance Hospital, Yonsei University College of Medicine, Seoul, South Korea; 3 Department of Radiology, Research Institute of Radiological Science, Severance Hospital, Yonsei University College of Medicine, 50-1 Yonsei-ro, Seodaemun-gu, Seoul, 03722 South Korea

**Keywords:** Cardiology, Medical research

## Abstract

The study aimed to develop and validate whether the computed tomography (CT) radiomics analysis is effective in differentiating cardiac tumors and thrombi. For this retrospective study, a radiomics model was developed on the basis of a training dataset of 192 patients (61.9 ± 13.3 years, 90 men) with cardiac masses detected in cardiac CT from January 2010 to September 2019. We constructed three models for discriminating between a cardiac tumor and a thrombus: a radiomics model, a clinical model, which included clinical and conventional CT variables, and a model that combined clinical and radiomics models. In the training dataset, the radiomics model and the combined model yielded significantly higher differentiation performance between cardiac tumors and cardiac thrombi than the clinical model (AUC 0.973 vs 0.870, p < 0.001 and AUC 0.983 vs 0.870, p < 0.001, respectively). In the external validation dataset with 63 patients (59.8 ± 13.2 years, 26 men), the combined model yielded a larger AUC compared to the clinical model (AUC 0.911 vs 0.802, p = 0.037). CT radiomics analysis is effective in differentiating cardiac tumors and thrombi. In conclusion, the combination of clinical, conventional CT, and radiomics features demonstrated an additional benefit in differentiating between cardiac tumor and thrombi compared to clinical data and conventional CT features alone.

## Introduction

Although cardiac tumors are rare, it is very important to diagnose them accurately due to various complications such as stroke, myocardial infarction, and arrhythmia^1^. In particular, the differential diagnosis of cardiac tumors and cardiac thrombi is essential for establishing treatment strategies^2,3^. Surgical resection is recommended for benign tumor such as cardiac myxoma or large papillary fibroelastoma, whereas cardiac thrombus can be managed with anticoagulation or thrombectomy^3,4^. Cardiac magnetic resonance imaging (CMR) has been widely used as an imaging modality of choice in patients with a cardiac mass^5^. However, the CMR process is hampered by high cost, long acquisition time, relatively low spatial resolution, and limited feasibility in unstable patients. Cardiac computed tomography (CT) can be an alternative imaging technique for evaluating cardiac masses, especially in patients with known contraindications for CMR or in patients with non-diagnostic images from other non-invasive methods. However, cardiac CT has not been widely used for this purpose because the tissue contrast of cardiac CT is inferior to that of CMR^6^. Also, additional non-contrast and delayed CT imaging can be required to distinguish cardiac tumors from thrombi^7^.

Radiomics, which is an emerging tool in the field of precision medicine, is a quantitative method of revealing associations between qualitative and quantitative information extracted from clinical images^8,9^. Radiomics converts clinical images such as CT scans into high-throughput quantitative data that can be used to improve diagnostic, prognostic, and predictive accuracy. The basic concept of the process is that both qualitative and quantitative information in clinical images may reflect the basic pathophysiology of the tissue^10^. Therefore, non-invasive and quantitative radiomic features can complement the information gained from tissue sampling and circulating biomarkers. Radiomics has mainly been applied to oncology studies, but recently it has gained increasing use for the diagnosis and assessment of prognosis of cardiovascular disease^11–17^. A recent study using CMR introduced the diagnostic potential of the radiomics score derived from native T1 maps to differentiate cardiac tumors from thrombi^18^.

This study aimed to develop a radiomics signature and validate whether a CT radiomics analysis is effective in differentiating cardiac tumors and thrombi. In addition, we evaluated whether the CT radiomics analysis could provide additional diagnostic performance to differentiate between a cardiac tumor and a thrombus compared to clinical and conventional CT features.

## Methods

### Ethical considerations

This retrospective multicenter study was approved by the Institutional Review Board of each center (Severance Hospital clinical trial center, Pusan National University Hospital clinical trial center and Gangnam Severance Hospital clinical trial center), and the requirement of obtaining informed consent was waived (Severance Hospital clinical trial center, Pusan National University Hospital clinical trial center and Gangnam Severance Hospital clinical trial center). All methods were performed in accordance with the relevant guidelines and regulations.

### Study design and patient selection

**Figure 1:**
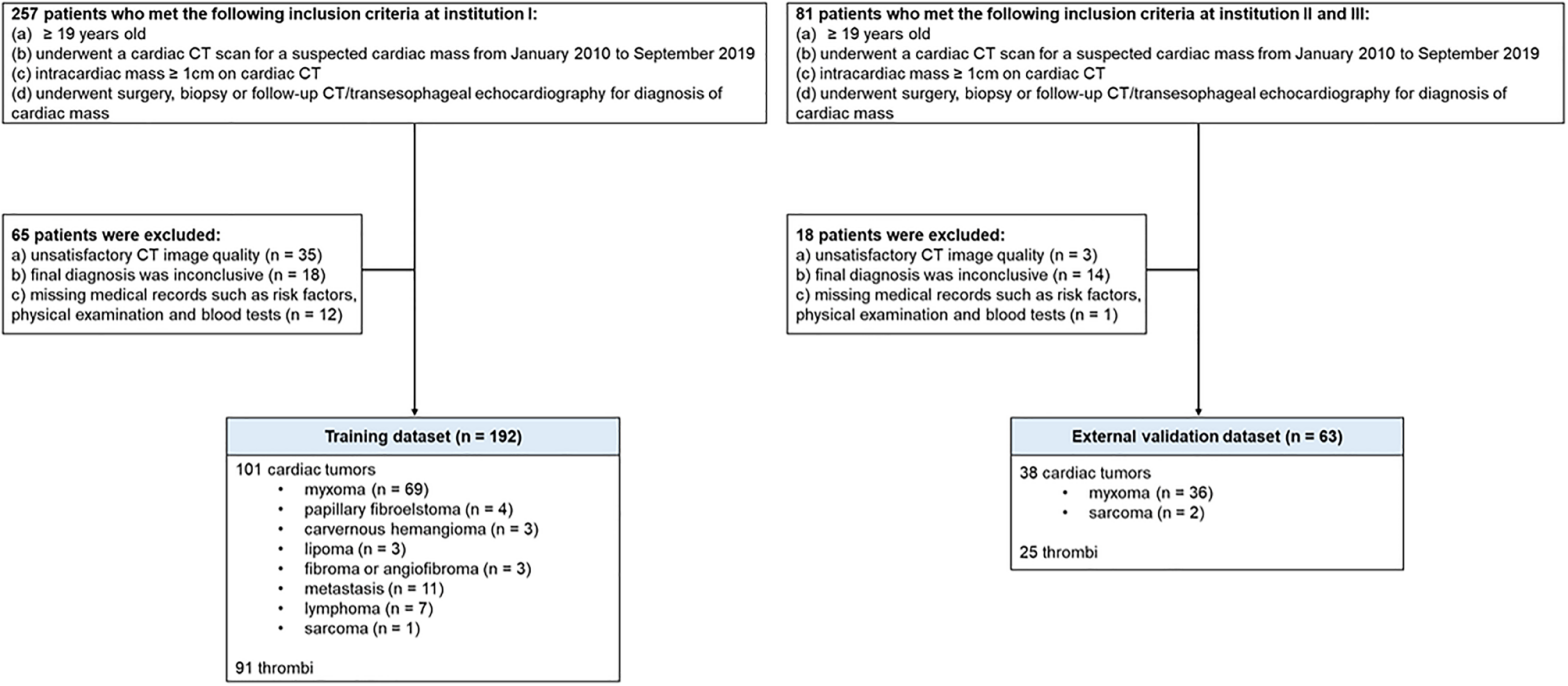
Patient selection flowchart.

In this multicenter study, a radiomic analysis was applied retrospectively to two independent data sets. For the training dataset, we retrospectively enrolled 257 consecutive patients who met the following inclusion criteria at each institution I: (1) ≥ 19 years old, (2) underwent a cardiac CT scan for a suspected cardiac mass from January 2010 to September 2019, (3) cardiac mass ≥ 1 cm on cardiac CT, and (4) underwent surgery, biopsy, or follow-up CT/transesophageal echocardiography for diagnosis of a cardiac mass. Among them, we excluded patients with unsatisfactory CT image quality (n = 35), patients whose final diagnosis was inconclusive (n = 18), or patients whose medical records such as risk factors, physical examination, and blood tests were missing (n = 12). Finally, a total of 192 patients (mean age of 61.9 ± 13.3 years, 90 men) with cardiac masses were included in the training dataset. For the external validation dataset, we retrospectively included 63 patients (mean age of 59.8 ± 13.2 years, 26 men) with cardiac masses who underwent cardiac CT from two independent institutes (Institution II and Institution III) with the same inclusion and exclusion criteria. (Fig. 1).

All data entry, data management, and analyses were coordinated or performed at a data-coordinating center. In our database, the following clinical and radiological characteristics were recorded: age, sex, history of cerebrovascular accident (CVA), atrial fibrillation or flutter, diabetes mellitus, hypertension, dyslipidemia and smoking, history of cardiac disease (including valvular heart disease, and congestive heart failure), histology subtypes, cardiac mass location, mass size, and mass density.

### Cardiac computed tomography (CT) examinations

All patients were required to be fasting for at least 4 h and abstain from caffeine at least 12 h before their CT examination. Beta blockers were administered in patients with high heart rates. All cardiac CT examinations were performed using a (at minimum) 64-slice multi-detector single- or dual-source CT scanner. The type of scanner used differed among institutions and included both single-source and dual-source scanners. For the training dataset, imaging was performed using the following CT scanners: SOMATOM Definition Flash, (Siemens, GmbH, Erlangen, Germany) and Revolution CT, (GE Healthcare, Waukesha, WI, USA). For the external validation dataset, imaging was performed using the following CT scanners: SOMATOM Definition, Revolution CT, and Brilliance 64 (Philips Healthcare, Cleveland, OH, USA). All CT images were acquired according to standardized scanning protocols (Supplementary Table 1) adapted to the equipment at each center. Axial CT images reconstructed with 0.625–0.9 mm slices and 0.45–0.625 mm intervals were used for image analysis.

### Image analysis

Image analyses were performed at the data-coordinating center by two experienced board-certificated cardiac radiologists with 8 and 11 years of experience in cardiac CT imaging who were blinded to the clinical and histologic findings. Conventional CT variables including cardiac mass location, mass size, mass density (defined by the Hounsfield unit [HU]) were recorded.

### CT radiomic feature extraction

**Figure 2:**
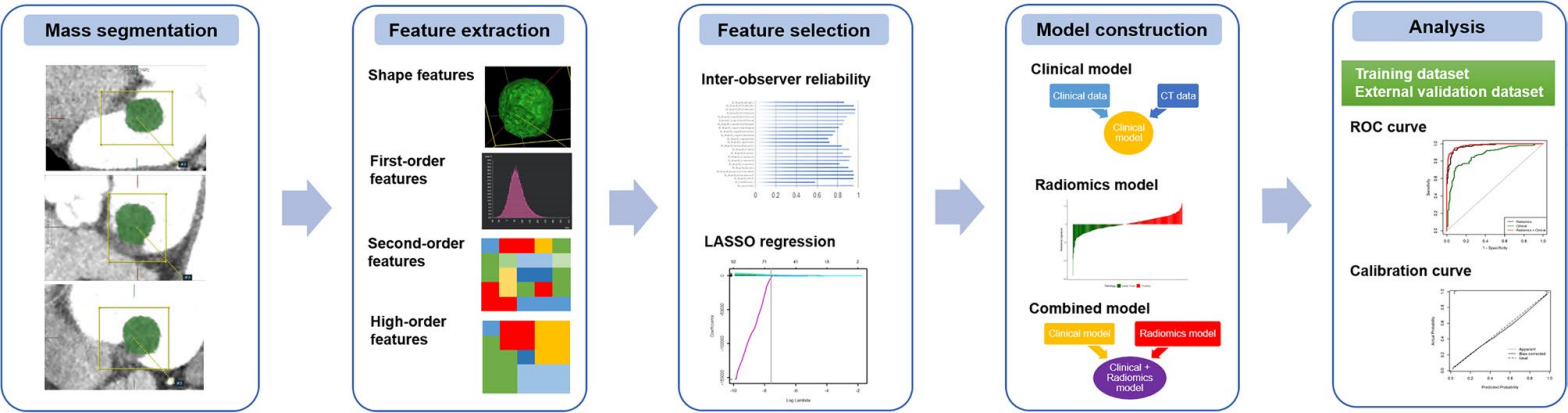
Flowchart showing the process of radiomics analysis. *LASSO* least absolute shrinkage and selection operator; *ROC* receiver operating characteristic.

The process of radiomics analysis is depicted in Fig. 2. Contrast enhanced cardiac CT images were first registered, followed by mass segmentation by two experienced board-certificated cardiac radiologists who were blinded to the clinical and histologic findings for the training dataset. A Digital Imaging and Communications in Medicine (DICOM) file was loaded to commercialized software (AVIEW, Coreline soft Inc., Seoul, South Korea) and lesion segmentation was performed for analysis. For quantitative analysis, the entire volume of interest (VOI) of the cardiac mass was delineated around the mass outline slice by slice on the axial CT images using the software. Specifically, after importing DICOM files into the software, we used brush tools to manually delineate the VOI slice-by-slice at the voxel level. Image magnification and 3-dimensional view techniques were used to facilitate precise segmentation. Feature extraction was performed using an AVIEW software package (Coreline Soft Co., Ltd., version 1.0.34.26) based on open source program for radiomic analysis, Pyradiomics (Pyradiomics library, version 2.2.0; Computational Imaging and Bioinformatics Lab, Harvard Medical School^19^. In this study, 127 radiomic features were extracted: 25 shape features, 31 first-order histogram features, 22s-order texture features, and 49 high-order features. For the external validation dataset, radiomic features were extracted in the same way by one radiologist.

### CT radiomic feature selection and model construction

Prior to feature selection and model building, no data transformation or standardization was conducted. Intraclass correlation coefficients based on a two-way mixed effect, were applied to perform a reproducibility assessment of CT radiomic features. The features with correlation coefficients > 0.75 were chosen for further analysis (Supplementary Table 2). Therefore, a total 98 of 127 features were selected as candidates for a least absolute shrinkage and selection operator (LASSO) analysis. The LASSO logistic regression model was used to select the appropriate radiomics features and build a classification model^20^. Ten-fold cross validation was performed to solve the overfitting. Features were selected if the mean of the calculated area under the curve (AUC) of the receiver operating characteristic (ROC) curve was equivalent to the maximum value. In addition, multivariable logistic regression was used to generate clinical and CT variables. Only variables with p < 0.05 in the univariable analyses were added to the final multivariable models to prevent model over-fitting. We constructed three models for discriminating between a cardiac tumor and a thrombus: a radiomics model, using CT radiomics variables; a clinical model, using clinical and conventional CT variables; a combined model, using clinical, conventional CT, and radiomics variables. Feature selection was performed only on the training dataset in order to maintain independence between the training and external validation datasets. The ROC curve was plotted to assess the differentiating performance of the three models with the training and external validation datasets.

### Statistical analysis

Categorical variables were compared by the chi-square or Fisher’s exact test. The differences of continuous variables were analyzed through the Student’s test or the Mann–Whitney U test. Demographics, CT results, and CT radiomic features were compared between cardiac tumor and thrombus groups. The best cutoff for the predicted probability of each model in the training set was based on the Youden index (when the sum of sensitivity and specificity becomes the maximum). The model performance was compared using the ROC curve of each model to identify the model with the higher predictability. Calibration curves were performed to assess the calibration of the radiomics model, accompanied with the Brier score and the Spiegelhalter z-test. To quantify the discrimination performance of the radiomics model, AUCs were calculated in training and external validation datasets. Obuchowski method and Delong method were used to compare the performance of the ROC curves^21,22^.

Values of p < 0.05 were considered statistically significant. All statistical analyses were completed by an R statistical package (version 3.6.3, R Foundation for Statistical Computing, Vienna, Austria). The packages named ‘caret’, ‘rms’, and ‘pROC’ were used for feature selection.

## Results

### Patient characteristics

The study included 192 (mean age of 61.9 ± 13.3 years, 90 men) and 63 patients (mean age of 59.8 ± 13.2 years, 26 men) in the training and external validation datasets, respectively. The number of patients who underwent surgery or biopsy in the training and external validation datasets were 112 and 39, respectively. The cardiac masses in the other patients (80 in the training set and 24 in the external validation set) were diagnosed as thrombi based on the patients’ response to anticoagulation treatment during follow-up. In the training dataset, there were 101 cardiac tumors and 91 thrombi in the 192 patients. Among the 101 cardiac tumors, 82 were benign, including myxoma (n = 69), papillary fibroelastoma (n = 4), cavernous hemangioma (n = 3), lipoma (n = 3), and fibroma or angiofibroma (n = 3). Nineteen were malignant, including metastasis (n = 11), lymphoma (n = 7) and sarcoma (n = 1). In the external dataset, there were 38 cardiac tumors and 25 thrombi in the 63 patients. Among the 38 cardiac tumors, 36 were myxoma and 2 were sarcoma. All tumors were confirmed by surgical excision or biopsy.

The baseline characteristics of all patients are summarized in Table 1. In the training dataset, patients with thrombi had higher incidences of history of CVA (p = 0.004), atrial fibrillation or flutter (p < 0.001), and history of cardiac disease (p < 0.001) than those with cardiac tumors. Other clinical characteristics of the two groups were not significantly different. The mean diameter of cardiac tumors was significantly larger than that of thrombi (35.7 ± 16.7 vs 27.7 ± 15.0 mm, p < 0.001). In the external validation dataset, patients with thrombi had higher incidences of a history of atrial fibrillation or flutter (p = 0.005) than those with cardiac tumors. The mean diameter of the cardiac tumors was significantly larger than that of thrombi (33.9 ± 15.2 vs 26.2 ± 13.7 mm, p = 0.045).

**Table 1:** Baseline characteristics of training and validation study datasets.

Characteristic	Training dataset			External validation dataset		
	Tumor	Thrombus	p value	Tumor	Thrombus	p value
	(n = 101)	(n = 91)		(n = 38)	(n = 25)	
Age	62.0 ± 13.8	61.9 ± 12.8	0.975	59.1 ± 13.0	60.5 ± 13.4	0.672
Sex (male)	45 (44.5)	45 (49.4)	0.592	14 (36.8)	12 (48.0)	0.53
**Clinical condition**						
Old CVA	4 (3.9)	16 (17.6)	0.004	3 (7.9)	6 (24.0)	0.156
History of cardiac disease^b^	3 (2.9)	22 (24.1)	< 0.001	4 (10.5)	8 (32.0)	0.072
Atrial fibrillation or flutter	11 (10.8)	50 (54.9)	< 0.001	3 (7.9)	10 (40.0)	0.005
Diabetes mellitus	23 (22.7)	23 (25.3)	0.8	6 (15.7)	6 (24.0)	0.621
Hypertension	38 (37.6)	41 (45.1)	0.364	14 (36.8)	8 (32.0)	0.903
Dyslipidemia	4 (3.9)	9 (9.9)	0.171	7 (18.4)	4 (16.0)	0.925
Smoking^a^	29 (28.7)	31 (34.1)	0.515	6 (15.7)	7 (28.0)	0.388
**Location**						
LA	68 (67.4)	60 (65.9)	0.947	24 (63.2)	14 (56.0)	0.758
LV	6 (5.9)	17 (18.7)	0.012	3 (7.9)	7 (28.0)	0.074
RA	22 (21.8)	14 (15.4)	0.342	10 (26.3)	4 (16.0)	0.531
RV	5 (4.9)	0 (0.0)	0.092	1 (2.6)	0 (0.0)	0.823
**CT measurement**						
Size (mm)	35.7 ± 16.7	27.7 ± 15.0	< 0.001	33.9 ± 15.2	26.2 ± 13.7	0.045
CT density (HU)	74.3 ± 25.7	69.2 ± 20.6	0.134	74.9 ± 26.2	58.7 ± 16.3	0.007

Values are presented as mean value (± standard deviation) or patient number (%).

*CT* computed tomography, *CVA* cerebrovascular accident, *HU* hounsfield unit, *LA* left atrium, *LV* left ventricle, *RA* right atrium, *RV* right ventricle.

^a^Current or former smoker.

^b^Cardiac disease includes valvular heart disease and congestive heart failure.

### Clinical and CT variables associated with cardiac tumor or thrombus

The univariable logistic regression analysis showed that the presence of old CVA (odds ratio (OR), 0.191; 95% confidence interval (CI), 0.061–0.604; p = 0.005), history of cardiac disease (OR, 0.100; 95% CI, 0.032–0.331; p < 0.001), atrial fibrillation or flutter (OR, 0.100; 95% CI, 0.052–0.213; p < 0.001), and location of left ventricle (LV) (OR, 0.132; 95% CI, 0.042–0.347; p < 0.001) was predictive of cardiac thrombi. In addition, larger mass (OR, 1.032; 95% CI, 1.013–1.062; p = 0.004) was significantly associated with a prediction of a cardiac tumor (Table 2). In the multivariable logistic regressions that were adjusted for age and sex, the presence of old CVA (OR, 0.291; 95% CI, 0.088–0.960; p = 0.043), history of cardiac disease (OR, 0.072; 95% CI, 0.022–0.236; p < 0.001), atrial fibrillation or flutter (OR, 0.053; 95% CI, 0.020–0.236; p < 0.001), and cardiac mass in the LV (OR, 0.121; 95% CI, 0.038–0.389; p < 0.001) were predictive of cardiac thrombi. In addition, larger mass (OR, 1.038; 95% CI, 1.038–1.074; p = 0.034) was significantly associated with the prediction of a cardiac tumor (Table 2).

**Table 2:** Univariable and multivariable analysis of training dataset for the clinical model (clinical and CT variables) predicting the cardiac tumor.

Characteristic	AUC of individual characteristics (95% CI)	Univariable analysis		Multivariable analysis	
		OR (95% CI)	p value	OR (95% CI)	p value
Age	0.510 (0.427–0.592)	1.0 (0.980–1.020)	0.975	1.019 (0.984–1.055)	0.300
Sex (male)	0.476 (0.405–0.547)	0.821 (0.475–1.452)	0.497	0.980 (0.466–2.061)	0.957
**Clinical condition**					
Old CVA	0.432 (0.388–0.476)	0.191(0.061–0.604)	0.005	0.291 (0.088–0.960)	0.043
History of cardiac disease	0.394 (0.347–0.441)	0.100 (0.032–0.331)	< 0.001	0.072 (0.022–0.236)	< 0.001
Atrial fibrillation or flutter	0.720 (0.660–0.780)	0.100 (0.052–0.213)	< 0.001	0.053 (0.020–0.236)	< 0.001
Diabetes mellitus	0.487 (0.427–0.548)	0.871 (0.451–1.692)	0.685		
Hypertension	0.463 (0.393–0.533)	0.743 (0.412–1.312)	0.297		
Dyslipidemia	0.470 (0.434–0.507)	0.382 (0.112–1.267)	0.114		
Smoking^a^	0.474 (0.407–0.541)	0.894 (0.607–1.304)	0.536		
**Location**					
LA	NA	1		1	
LV	NA	0.132 (0.042–0.347)	< 0.001	0.121 (0.038–0.389)	< 0.001
RA	NA	0.531 (0.240–1.213)	0.131		
RV	NA	3.772 (0.412–502.4)	0.292		
**CT measurement**					
Size (mm)	0.647 (0.571–0.723)	1.032 (1.013–1.062)	0.004	1.038 (1.003–1.074)	0.034
CT density (HU)	0.536 (0.457–0.614)	1.0 (0.991–1.012)	0.831		

Values are presented as mean value (± standard deviation) or patient number (%).

*AUC* Area under the curve, NA not applicable.

^a^Current or former smoker.

### Model performance and validation

**Figure 3:**
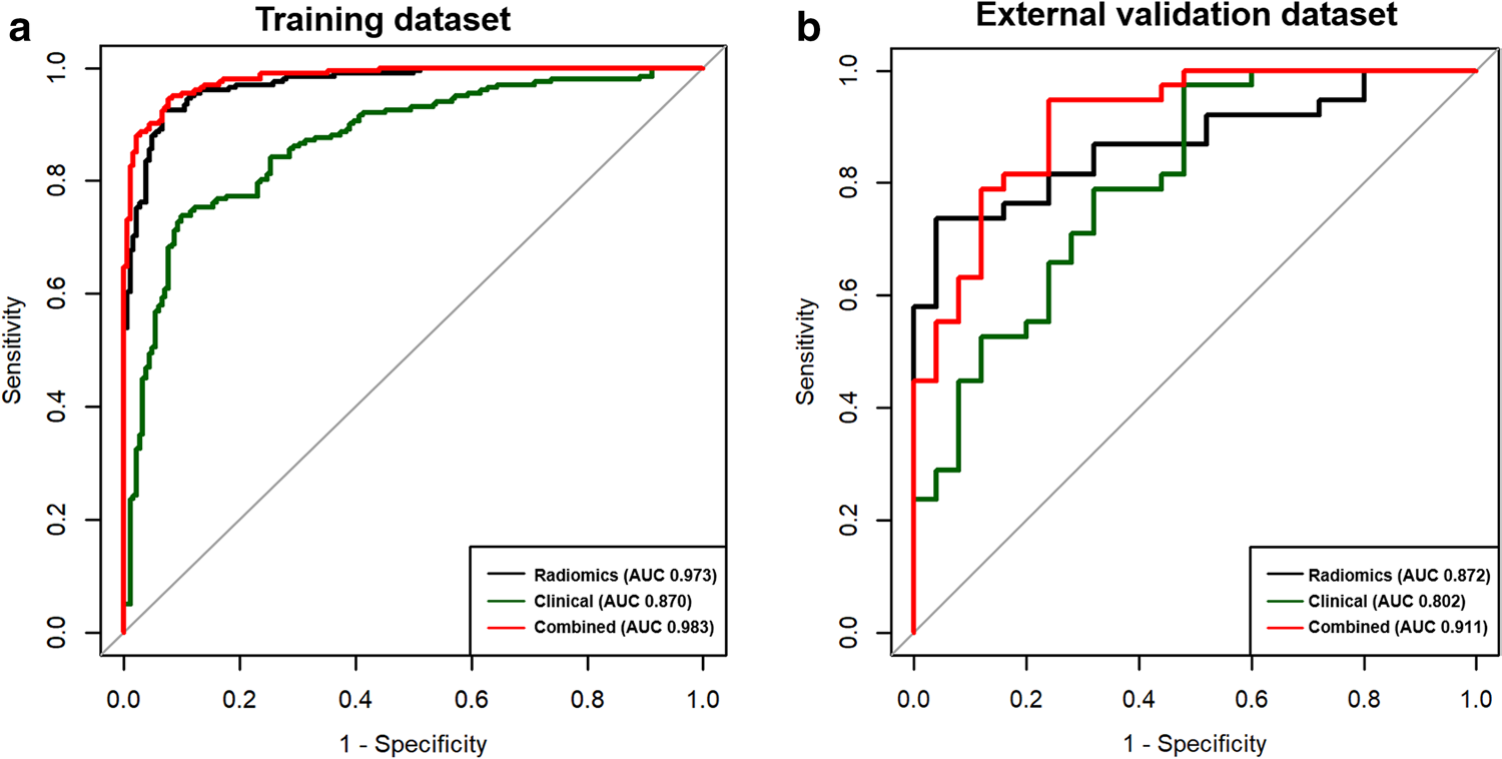
ROC curve of the radiomics model, clinical model, and combined model with radiomics and clinical model to predict cardiac tumor in different datasets. (**a**) The training dataset. (**b**) The external validation dataset.

**Figure 4:**
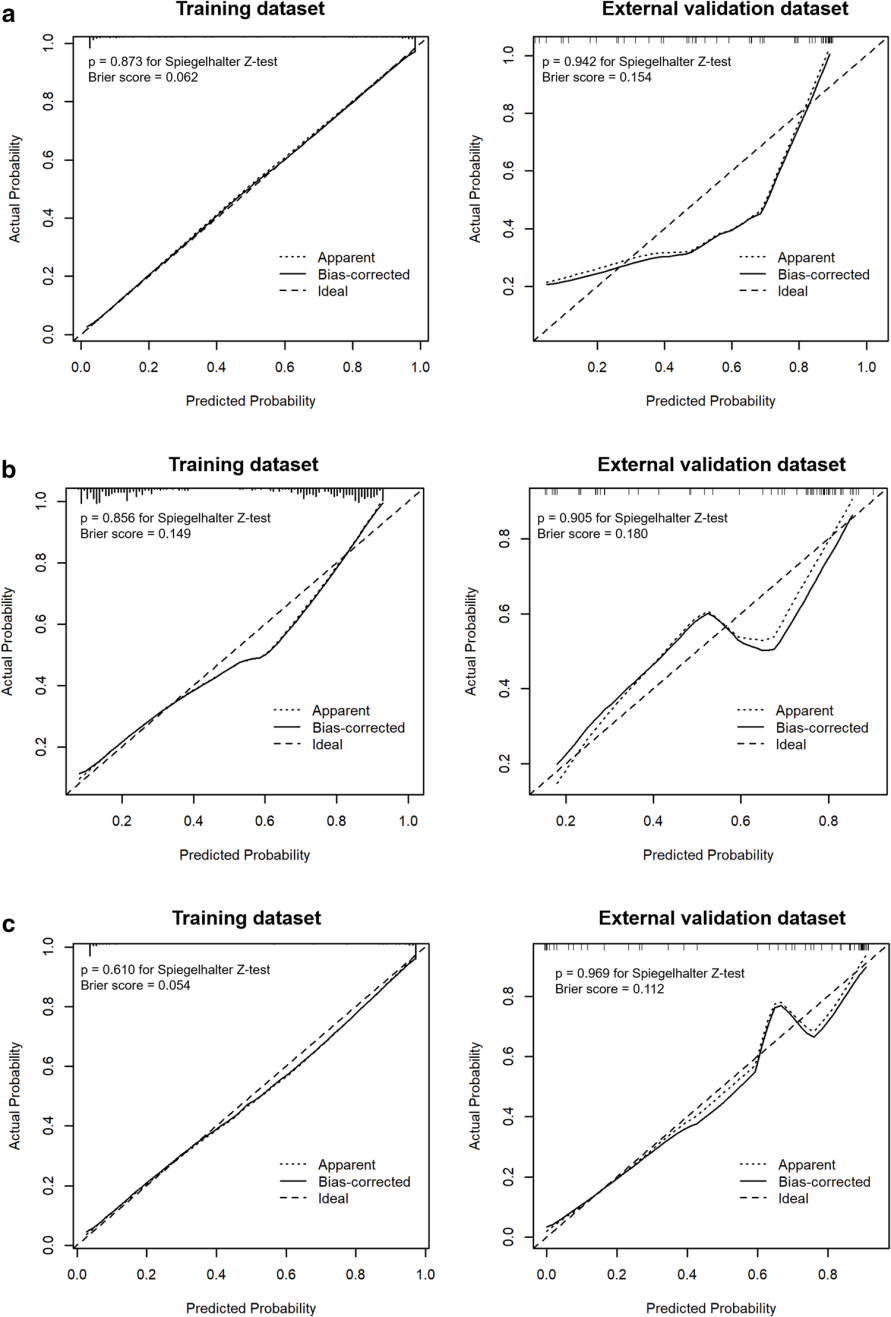
Calibration curves of the different models in the training and validation datasets. (**a**) The radiomics model. (**b**) The clinical model. (**c**) The combined model.

Of the 127 radiomics features, the 98 most reproducible features (interobserver intraclass correlation coefficient values > 0.75) were selected for subsequent analysis. Using LASSO regression, a total of 64 features (Supplementary Table 3) were selected to construct a radiomics model for discriminating cardiac tumors and cardiac thrombi. Table 3 and Fig. 3 demonstrate the discrimination performance of the radiomics, clinical, and the combined (radiomics plus clinical model) models. In the training dataset, the radiomics model showed good discriminatory performance with an AUC of 0.973 [95% CI, 0.956–0.989]. Using optimal cutoff values of 0.507, 0.684, and 0.488, diagnostic accuracy of the radiomics, clinical, and combined models were 93.0%, 81.5%, and 93.5%, respectively. The radiomics and combined models yielded significantly higher discriminating performance between cardiac tumors and thrombi than the clinical model (AUC 0.973 vs 0.870, p < 0.001 and accuracy 93.5% vs 81.5, p < 0.001; AUC 0.983 vs 0.870, p < 0.001 and accuracy 93.5% vs 81.5%, p < 0.001, respectively). Combined model yielded higher discriminating performance than radiomics model alone (AUC 0.983 vs AUC 0.973, p = 0.022). In the external validation dataset, the combined model yielded the largest AUC of 0.911 [95% CI, 0.839–0.982], which supported the improved discriminating performance compared to the clinical model (AUC 0.911 vs 0.802, p = 0.037). Sensitivity and accuracy of the radiomics model and the combined model was significantly higher than those of the clinical model. However, the difference of the differentiation performance between the radiomics model and the clinical model was not statistically significant (AUC 0.872 vs 0.802, p = 0.331). The calibration curves of the combined model showed good calibration for predictive probabilities, with a non-significant goodness of fit in the Spiegelhalter z-test for both the training dataset (p = 0.610) and the validation dataset (p = 0.969) (Fig. 4).

**Table 3:** Discrimination performance of radiomics, clinical, and combined (radiomics plus clinical model) models in training and validation datasets.

Dataset		Models			p value comparison of models		
		Radiomics model	Clinical model	Combined model	Radiomics vs clinical	Radiomics vs combined	Clinical vs combined
Training	AUC (95% CI)	0.973 (0.956–0.989)	0.870 (0.820–0.921)	0.983 (0.971–0.995)	< 0.001	0.022	< 0.001
	Sensitivity (%)	92.6	73.8	94.6	< 0.001	0.153	< 0.001
	Specificity (%)	93.4	90.1	92.3	0.315	0.478	0.515
	Accuracy (%)	93.0	81.5	93.5	< 0.001	0.617	< 0.001
External validation	AUC (95% CI)	0.872 (0.786–0.958)	0.802 (0.690–0.915)	0.911 (0.839–0.982)	0.331	0.342	0.037
	Sensitivity (%)	95.5	71.1	100.0	< 0.001	0.426	< 0.001
	Specificity (%)	78.0	72.0	81.3	0.229	0.321	0.069
	Accuracy (%)	85.3	71.4	89.4	0.021	0.407	0.004

*AUC* area under the curve, *NA* not applicable.

**Figure 5:**
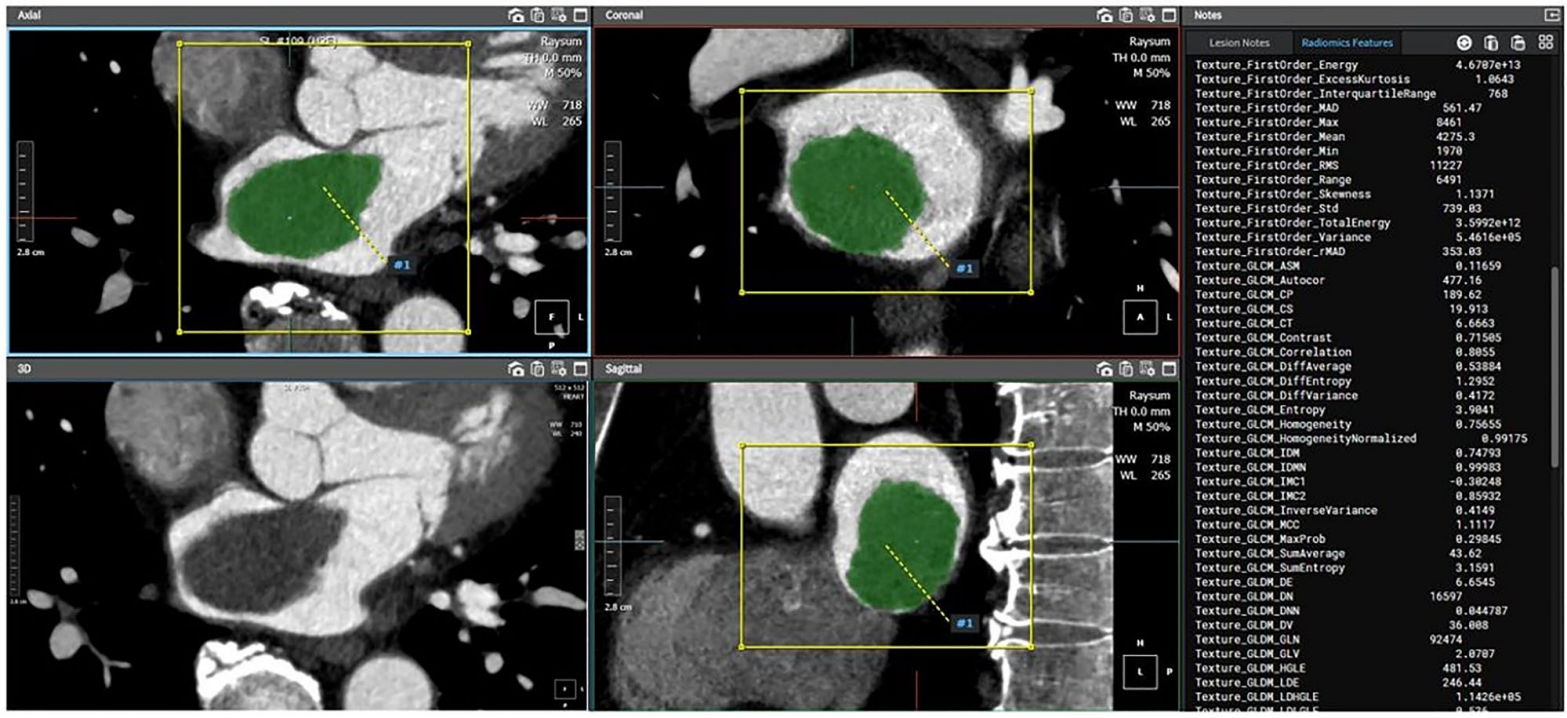
A 67-year-old man with cardiac thrombus in the left ventricle. A lobulated mass (25 mm) in the left ventricle. The predictive probability of tumor based on radiomics features of this mass was 0.237.

**Figure 6:**
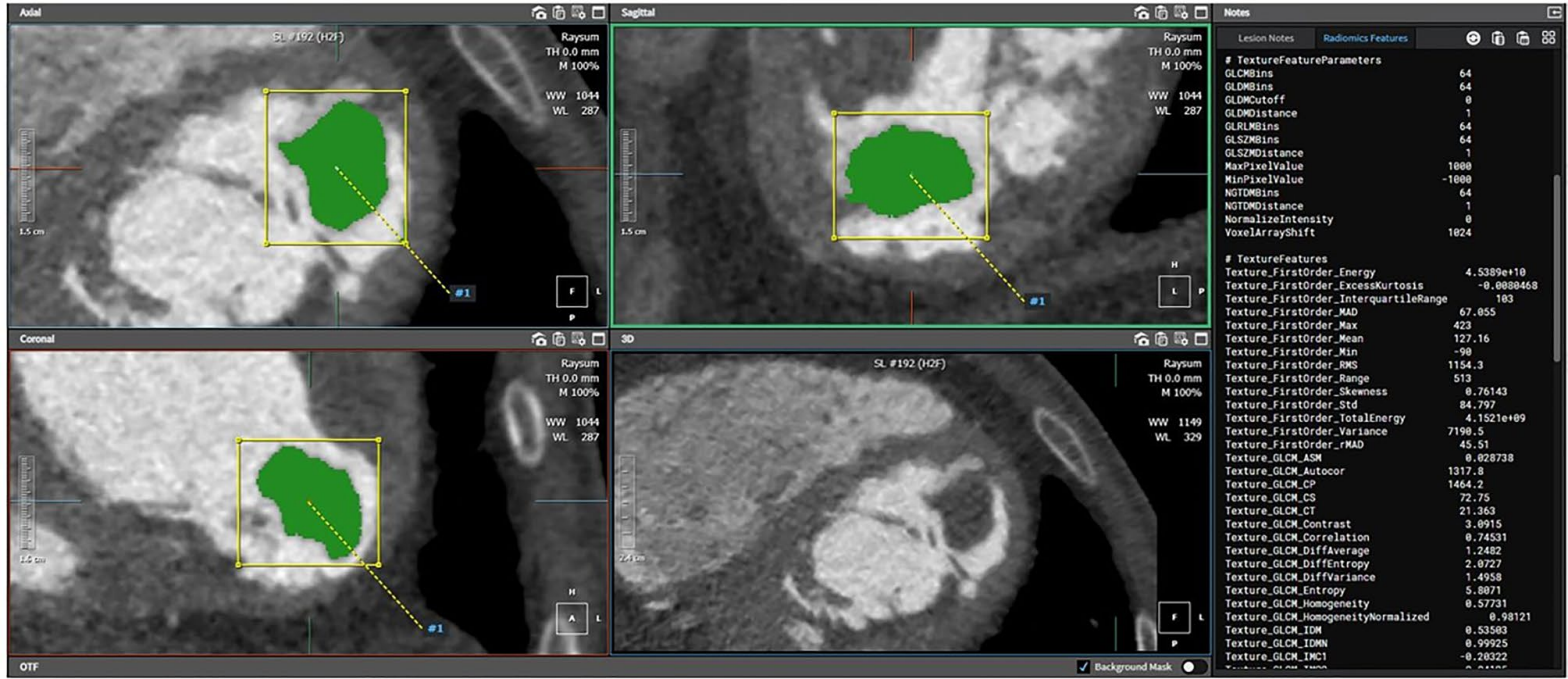
A 53-year-old man with cardiac myxoma in the left atrium. A lobulated mass (43 mm) in the left atrium. The predictive probability of tumor based on radiomics features of this mass was 0.821.

Tables 4 and 5 demonstrated the multivariable analysis for the conventional CT model (size and CT density) predicting the cardiac tumor in the training dataset and the discrimination performance of the radiomics and conventional CT model (size and CT density). In the training dataset, radiomics model yielded significantly higher discriminating performance between cardiac tumors and thrombi than the conventional CT model (AUC 0.973 vs 0.652, p < 0.001). However, the differentiation performance between the radiomics model and the conventional CT model was not statistically significant (AUC 0.872 vs 0.753, p = 0.122) in the external validation dataset. (Figs. 5 and 6).

**Table 4:** Multivariable analysis of training dataset for the conventional CT model (size and CT density) predicting the cardiac tumor.

Characteristic	Adjusted OR (95% CI)	p value
Size (mm)	1.035 (1.012–1.058)	0.003
CT density (HU)	1.004 (0.993–1.016)	0.426

**Table 5:** Discrimination performance of radiomics and conventional CT models in training and validation datasets.

Dataset	Models	AUC (95% CI)	p value comparison of models
Training	Radiomics model	0.973 (0.956–0.989)	< 0.001
	Conventional CT model	0.652 (0.576–0.728)	
External validation	Radiomics model	0.872 (0.786–0.958)	0.122
	Conventional CT model	0.753 (0.625–0.880)

## Discussion

This study was designed to develop and validate whether the CT radiomics analysis technique is effective in differentiating cardiac tumors and thrombi. The main finding was that a combination of clinical, conventional CT, and radiomics features demonstrated additional benefits in differentiating between cardiac tumors and thrombi compared to clinical model (clinical and conventional CT features) alone.

Distinguishing a cardiac thrombus from a tumor is challenging because the clinical and radiological signs are very similar, but the subsequent medical treatment is different. CMR is currently the standard method for evaluating cardiac mass^5^. Previous studies have reported that late gadolinium enhancement-CMR can be useful for differentiating between cardiac tumors and thrombi^23–25^.

Cardiac CT has been proposed to help differentiate between cardiac tumors and thrombi as part of a multimodality approach. Although useful imaging findings of cardiac tumors and thrombi can be found on CT scans, the potential overlap in the imaging findings often may result in persisting uncertainty in the differentiation of the two diseases. Previous studies have used only visual assessment or HU–based CT values to differentiate between cardiac tumors and thrombi in CT^26–28^. The problem with these analyses is moderate inter-reader reproducibility of visual assessment and only rely on the distribution of CT numbers, which shows major overlaps between two disease entities. In our present study, a larger cardiac mass (OR, 1.09; 95% CI, 1.04–1.07; p = 0.034) was significantly associated with the prediction of a cardiac tumor, while the CT-based HU value was not predictive for either cardiac tumor or thrombus. As different disease entities require different management strategies, it is crucial to obtain a correct diagnosis in the most noninvasive way as possible.

Radiomics is an emerging field of study that allows to extract quantitative imaging features from radiological datasets and describe the heterogeneity and spatial complexity of given regions of interest. This feature generative technique makes it possible to precise identification of phenotype abnormalities in medical images and may provide additional information, potentially allowing histological classification of abnormalities based on the images^29^. In this study, we constructed three models for discriminating between cardiac tumor and cardiac thrombus and compared the model performance in testing and validation datasets. Our radiomics model showed good discriminatory performance between cardiac tumors and thrombi with AUC of 0.973 and 0.872 in training and validation datasets, respectively. When we combined the radiomics model with the clinical model, our combined model showed significantly higher discriminating performance between cardiac tumors and thrombi than that of the clinical model (clinical and conventional CT parameters) in an external validation dataset (AUC 0.911 vs 0.802, p = 0.037). In both training and external datasets, sensitivity and diagnostic accuracy of combined models were higher than those of clinical model. This result validated and supported that radiomics may have additional value in the differentiation between cardiac tumors and thrombi using CT. Recent investigation demonstrated that when histological classification of coronary lesions can be predicted using a radiomics-based machine learning model, which outperformed visual assessment^30,31^. It seems that radiomics can extract new information from medical images, which can improve the discriminatory power of current medical devices.

Despite the novel analysis in this study, several limitations must be acknowledged. First, the proposed radiomics model was established on the basis of data obtained from a single center. Although our radiomics model was validated with an external validation dataset, prospective multicenter studies with considerably large datasets are needed to further validate the robustness and reproducibility of our radiomics analysis. Second, the CT protocol can influence the results of the radiomics approach. In our study, image standardization was not performed when constructing the CT radiomics model. For CT radiomics, image reconstruction algorithms (i.e., reconstruction kernels) and section thickness have been major sources of radiomic feature variability^32,33^. A previous study demonstrated that different kernels significantly reduced the reproducibility of radiomic features, with only 15.2% of radiomic features were reliable when using different reconstruction kernel^33^. Although the CT images were not standardized, the results of our radiomics model improved the ability to differentiate between cardiac tumors and thrombi in external validation datasets with different CT images.

In conclusion, CT radiomics analysis is effective in differentiating cardiac tumors and thrombi. The combination of clinical, conventional CT, and radiomics features demonstrated additional benefits in differentiating between cardiac tumors and thrombi compared to clinical and conventional CT features alone. Hence, the combined model may be useful to differentiate between cardiac tumors and thrombi when other imaging modalities are inconclusive.

## Supplementary Information

Supplementary Tables.
