# Supplementary material for: CT-based radiomics signature for differentiation between cardiac tumors and thrombi: a retrospective, multicenter study
Source: Sci Rep. 2022 May 17;12:8173. doi: 10.1038/s41598-022-12229-x (PMC9114026; doi:10.1038/s41598-022-12229-x)
Supplement: Supplementary file 1 — Supplementary Tables. [file 41598_2022_12229_MOESM1_ESM.docx]

**Supplementary table 1.** Acquisition parameters and protocols for cardiac computed tomography (CT)

| **Imaging protocol** | **Somatom Definition**  **(n = 11)** | **Somatom Definition Flash**  **(n = 157)** | **Ingenuity Core 128**  **(n = 45)** | **Revolution CT**  **(n = 42)** |
| --- | --- | --- | --- | --- |
| **Detector channels** | 64 | 128 | 64 | 256 |
| **Gantry rotation time (ms)** | 330 | 280 | 400 | 280 |
| **Tube voltage (kVp)** | 100-120 | 100-120 | 100-120 | 100-120 |
| **Tube current-time (mAs)** | Automated dose modulation | Automated dose modulation | Automated dose modulation | Automated dose modulation |
| **ECG gating technique** | Prospective or retrospective | Prospective or retrospective | Prospective or retrospective | Prospective or retrospective |
| **Reconstructed slice thickness (mm)** | 0.75 | 0.75 | 0.9 | 0.625 |
| **Incremental interval (mm)** | 0.5 | 0.5 | 0.45 | 0.625 |
| **Reconstruction kernel** | B36f | B36f | XCC | Standard |
| **Contrast media** |  |  |  |  |
| **Concentration (mg iodine)** | 370 | 370 | 350 | 370 |
| **Flow rate (mL/s)** | 4-5 | 4-5 | 4-5 | 4-5 |
| **Amount (mL)** | 60-80 | 60-80 | 1mL/kg | 70-80 |

**Supplementary table 2.** Intra-class correlation coefficients (ICC) of the radiomics features.

| **Radiomics features** | **ICC (95% CI)** |
| --- | --- |
| 3D measure value | 0.953 (0.947–0.961) |
| 3D fractal dimension | 0.578 (0.442–0.636) |
| 3D shape volume | 0.953 (0.946–0.958) |
| 3D shape surface area | 0.959 (0.947–0.962) |
| 3D shape surface area to volume ratio | 0.950 (0.939–0.963) |
| 3D shape sphericity | 0.902 (0.885–0.917) |
| 3D shape compactness | 0.819 (0.775–0.833) |
| 3D shape compactness2 | 0.912 (0.901–0.930) |
| 3D shape compactness3 | 0.933 (0.919–0.945) |
| 3D shape roundness | 0.847 (0.823–0.856) |
| 3D shape circularity | 0.912 (0.901–0.930) |
| 3D shape spherical disproportion | 0.842 (0.831–0.885) |
| 3D shape longest 1st axis | 0.722 (0.680–0.778) |
| 3D shape longest 2nd axis | 0.709 (0.670–0.777) |
| 3D shape longest 1st axis on axial | 0.755 (0.733–0.807) |
| 3D shape longest 2nd axis on axial | 0.778 (0.735–0.806) |
| 3D shape longest 1st axis on sagittal | 0.811 (0.775–0.829) |
| 3D shape longest 2nd axis on sagittal | 0.852 (0.839–0.881) |
| 3D shape longest 1st axis on coronal | 0.862 (0.845–0.883) |
| 3D shape longest 2nd axis on coronal | 0.888 (0.862–0.891) |
| 3D shape PCA 1st major std | 0.966 (0.960–0.969) |
| 3D shape PCA 2nd major std | 0.971 (0.968–0.978) |
| 3D shape PCA 3rd major std | 0.958 (0.946–0.959) |
| 3D shape elongation | 0.863 (0.844–0.880) |
| 3D shape flatness | 0.846 (0.823–0.855) |
| 3D texture first order energy | 0.953 (0.949–0.958) |
| 3D texture first order total energy | 0.953 (0.947–0.959) |
| 3D texture first order min | 0.468 (0.452–0.479) |
| 3D texture first order max | 0.764 (0.740–0.786) |
| 3D texture first order range | 0.763 (0.723–0.776) |
| 3D texture first order mean | 0.956 (0.944–0.964) |
| 3D texture first order MAD | 0.836 (0.822–0.859) |
| 3D texture first order rMAD | 0.869 (0.843–0.88) |
| 3D texture first order RMS | 0.954 (0.930–0.957) |
| 3D texture first order std | 0.758 (0.736–0.772) |
| 3D texture first order skewness | 0.279 (0.204–0.413) |
| 3D texture first order excess kurtosis | 0.194 (0.051–0.313) |
| 3D texture first order variance | 0.698 (0.674–0.713) |
| 3D texture first order interquartile range | 0.884 (0.864–0.899) |
| 3D texture histogram mean | 0.956 (0.945–0.965) |
| 3D texture histogram std | 0.787 (0.767–0.803) |
| 3D texture histogram kewness | 0.476 (0.401–0.537) |
| 3D texture histogram excess kurtosis | 0.294 (0.185–0.386) |
| 3D texture histogram energy | 0.857 (0.835–0.873) |
| 3D texture histogram entropy | 0.842 (0.820–0.858) |
| 3D texture histogram min | 0.468 (0.452–0.479) |
| 3D texture histogram max | 0.913 (0.886–0.933) |
| 3D texture histogram voxel count | 0.955 (0.951–0.958) |
| 3D texture percentile 10 | 0.973 (0.963–0.980) |
| 3D texture percentile 25 | 0.973 (0.963–0.981) |
| 3D texture percentile 50 | 0.956 (0.945–0.964) |
| 3D texture percentile 75 | 0.944 (0.932–0.953) |
| 3D texture percentile 90 | 0.908 (0.894–0.918) |
| 3D texture percentile 95 | 0.877 (0.862–0.887) |
| 3D texture gradient mean | 0.928 (0.905–0.945) |
| 3D texture gradient std | 0.815 (0.788–0.836) |
| 3D texture GLCM ASM | 0.904 (0.886–0.917) |
| 3D texture GLCM IDM | 0.969 (0.958–0.978) |
| 3D texture GLCM IDMN | 0.928 (0.903–0.948) |
| 3D texture GLCM homogeneity | 0.969 (0.957–0.977) |
| 3D texture GLCM homogeneity normalized | 0.956 (0.940–0.969) |
| 3D texture GLCM inverse variance | 0.935 (0.920–0.946) |
| 3D texture GLCM contrast | 0.928 (0.903–0.947) |
| 3D texture GLCM correlation | 0.826 (0.792–0.853) |
| 3D texture GLCM autocor | 0.948 (0.933–0.959) |
| 3D texture GLCM entropy | 0.904 (0.885–0.918) |
| 3D texture GLCM CP | 0.532 (0.512–0.547) |
| 3D texture GLCM CS | 0.515 (0.492–0.533) |
| 3D texture GLCM CT | 0.754 (0.725–0.776) |
| 3D texture GLCM sum entropy | 0.851 (0.828–0.869) |
| 3D texture GLCM diff average | 0.955 (0.939–0.968) |
| 3D texture GLCM diff entropy | 0.935 (0.919–0.948) |
| 3D texture GLCM diff variance | 0.887 (0.861–0.907) |
| 3D texture GLCM IMC1 | 0.874 (0.851–0.892) |
| 3D texture GLCM IMC2 | 0.813 (0.776–0.842) |
| 3D texture GLCM MCC | 0.597 (0.486–0.694) |
| 3D texture GLCM max prob | 0.947 (0.933–0.957) |
| 3D texture GLCM sum average | 0.956 (0.940–0.908) |
| 3D texture GLRLM SRE | 0.275 (0.122–0.394) |
| 3D texture GLRLM LRE | 0.446 (0.390–0.488) |
| 3D texture GLRLM LGRE | 0.369 (0.256–0.455) |
| 3D texture GLRLM HGRE | 0.301 (0.165–0.405) |
| 3D texture GLRLM SRLGE | 0.328 (0.195–0.433) |
| 3D texture GLRLM SRHGE | 0.250 (0.107–0.357) |
| 3D texture GLRLM LRLGE | 0.470 (0.431–0.50) |
| 3D texture GLRLM LRHGE | 0.439 (0.345–0.512) |
| 3D texture GLRLM GNUN | 0.316 (0.192–0.428) |
| 3D texture GLRLM RLNUN | 0.301 (0.176–0.412) |
| 3D texture GLRLM RP | 0.323 (0.178–0.437) |
| 3D texture GLRLM RV | 0.491 (0.444–0.525) |
| 3D texture GLRLM RE | 0.296 (0.137–0.416) |
| 3D texture GLRLM GLV | 0.277 (0.118–0.386) |
| 3D texture GLSZM SAE | 0.945 (0.931–0.956) |
| 3D texture GLSZM LAE | 0.972 (0.964–0.979) |
| 3D texture GLSZM GLN | 0.974 (0.972–0.975) |
| 3D texture GLSZM GLNN | 0.792 (0.771–0.809) |
| 3D texture GLSZM SZN | 0.948 (0.934–0.959) |
| 3D texture GLSZM SZNN | 0.965 (0.951–0.976) |
| 3D texture GLSZM ZP | 0.970 (0.957–0.970) |
| 3D texture GLSZM GLV | 0.946 (0.931–0.958) |
| 3D texture GLSZM ZV | 0.980 (0.970–0.987) |
| 3D texture GLSZM ZE | 0.782 (0.749–0.807) |
| 3D texture GLSZM LGLZE | 0.945 (0.931–0.956) |
| 3D texture GLSZM HGLZE | 0.972 (0.964–0.979) |
| 3D texture GLSZM SALGLE | 0.938 (0.922–0.951) |
| 3D texture GLSZM LAHGLE | 0.978 (0.970–0.984) |
| 3D texture NGTDM coarseness | 0.974 (0.965–0.982) |
| 3D texture NGTDM contrast | 0.785 (0.726–0.833) |
| 3D texture NGTDM busyness | 0.923 (0.894–0.945) |
| 3D texture NGTDM Complexity | 0.828 (0.776–0.870) |
| 3D texture NGTDM strength | 0.357 (0.351–0.362) |
| 3D texture GLDM SDE | 0.949 (0.933–0.961) |
| 3D texture GLDM LDE | 0.986 (0.978–0.992) |
| 3D texture GLDM GLN | 0.979 (0.977–0.98) |
| 3D texture GLDM DN | 0.952 (0.945–0.957) |
| 3D texture GLDM DNN | 0.977 (0.962–0.988) |
| 3D texture GLDM GLV | 0.741 (0.708–0.767) |
| 3D texture GLDM DV | 0.985 (0.975–0.993) |
| 3D texture GLDM DE | 0.802 (0.774–0.823) |
| 3D texture GLDM LGLE | 0.970 (0.955–0.982) |
| 3D texture GLDM HGLE | 0.949 (0.934–0.96) |
| 3D texture GLDM SDLGLE | 0.949 (0.942–0.955) |
| 3D texture GLDM SDHGLE | 0.932 (0.907–0.951) |
| 3D texture GLDM LDLGLE | 0.987 (0.973–0.997) |
| 3D texture GLDM LDHGLE | 0.984 (0.981–0.987) |
| 3D texture moment J1 | 0.955 (0.927–0.975) |
| 3D texture moment J2 | 0.917 (0.906–0.925) |

*PCA, Principal component analysis; MAD, Maximal absolute deviation; rMAD, Robust mean absolute deviation; RMS, Root mean square; GLCM, Grey level co-occurrence matrix; GLRLM, Gray level run length matrix; GLSZM, Gray level size zone matrix; NGTDM, Neighborhood gray tone difference matrix; GLDM, Gray level dependence matrix.*

**Supplementary table 3.** Selected features

| **Radiomics features** |
| --- |

3D fractal dimension

3D shape volume

3D shape surface area

3D shape surface area to volume ratio

3D shape compactness

3D shape compactness3

3D shape roundness

3D shape spherical disproportion

3D shape longest 1st axis

3D shape longest 2nd axis

3D shape longest 1st axis on axial

3D shape longest 2nd axis on axial

3D shape longest 1st axis on sagittal

3D shape longest 2nd axis on sagittal

3D shape longest 1st axis on coronal

3D shape longest 2nd axis on coronal

3D shape PCA 1st major std

3D shape PCA 2nd major std

3D shape PCA 3rd major std

3D shape elongation

3D shape flatness

3D texture first order energy

3D texture first order total energy

3D texture first order min

3D texture first order RMS

3D texture first order variance

3D texture first order interquartile range

3D texture histogram entropy

3D texture histogram min

3D texture histogram max

3D texture percentile 50

3D texture grad mean

3D texture gradient std

3D texture GLCM inverse variance

3D texture GLCM correlation

3D texture GLCM CP

3D texture GLCM CS

3D texture GLCM diff variance

3D texture GLCM IMC2

3D texture GLCM max prob

3D texture GLRLM LRLGE

3D texture GLRLM RV

3D texture GLSZM GLN

3D texture GLSZM GLNN

3D texture GLSZM SZN

3D texture GLSZM SZNN

3D texture GLSZM ZV

3D texture GLSZM ZE

3D texture GLSZM SALGLE

3D texture NGTDM coarseness

3D texture NGTDM contrast

3D texture NGTDM busyness

3D texture NGTDM complexity

3D texture NGTDM strength

3D texture GLDM DN

3D texture GLDM DNN

3D texture GLDM GLV

3D texture GLDM DE

3D texture GLDM SDLGLE

3D texture GLDM SDHGLE

3D texture GLDM LDLGLE

3D texture GLDM LDHGLE

3D texture moment J1

3D texture moment J2

*PCA, Principal component analysis; RMS, Root mean square; GLCM, Grey level co-occurrence matrix; GLRLM, Gray level run length matrix; GLSZM, Gray level size zone matrix; NGTDM, Neighborhood gray tone difference matrix; GLDM, Gray level dependence matrix.*
